# Uplift-driven sediment redness decrease at ~16.5 Ma in the Yumen Basin along the northeastern Tibetan Plateau

**DOI:** 10.1038/srep29568

**Published:** 2016-07-14

**Authors:** Weitao Wang, Peizhen Zhang, Wenjun Zheng, Dewen Zheng, Caicai Liu, Hongyan Xu, Huiping Zhang, Jingxing Yu, Jianzhang Pang

**Affiliations:** 1 State Key Laboratory of Earthquake Dynamics, Institute of Geology, China Earthquake Administration, Beijing, China; 2 School of Earth Science and Geological Engineering, Sun Yat-sen University, Guangzhou, China

## Abstract

Significant climate shifts in the northeastern Tibetan Plateau have taken place during the Cenozoic, but the reasons behind them remain unclear. In order to unravel the mechanisms driving these climate changes, proxy data with accurate age constraint are needed. Here we present magnetostratigraphy, sediment color (redness a*, and lightness L*) and grain-size analysis from an early to middle Miocene (~20–15.3 Ma) sediment sequence preserved in the Yumen Basin on the northeastern Tibetan Plateau. In this basin, remarkable increase in lightness, decreases in redness and in ratio of hematite (Hm) to goethite (Gt) took place at ~16.5 Ma. We suggest that these changes result from shorter duration of weathering, climatic wetting, and cooling associated with rapid uplift of the Qilian Shan at the middle Miocene.

The climate at the northeastern margin of the Tibetan Plateau changed significantly during the Cenozoic era^1^,^2^,^3^,^4^. According to previous studies, three main hypotheses have been proposed as the major forcing mechanisms of the climate shifts in the northeastern Tibetan Plateau. (1) The uplift of the Tibetan Plateau, which creates high topography in Asia, may affect northern hemisphere climate by increasing chemical weathering and its atmospheric thermal and blocking effects^4^,^5^. Locally, the emergence of mountain ranges may lead to the development of rain shadows where rainfall increases on the windward side of the ranges and decreases on the leeward side^6^. (2) Global cooling reduces the amount of precipitation in the atmosphere and forces the cool and dry climate in the region^7^. (3) The retreat of shallow Paratethys Sea once extending over Eurasia reduced precipitation in Central Asia and caused the aridification of this region during the early Cenozoic^8^,^9^. Given the complexity of the climate history in the northeastern Tibet, it seems likely that different driving forces may have operated at various periods but the details remain unclear.

A clear understanding of Eastern and Central Asian climate history and forcing mechanisms relies on the availability of well-dated Cenozoic climatic records in the northeastern Tibetan Plateau, where continuous basin deposits are particularly sensitive to regional uplift.

Sediment color is a useful tool for identification of sedimentary facies and qualitative determinations of paleoclimate^10^,^11^. Recently, soil color studies of loess-paleosol sequences from the Chinese Loess Plateau have been completed^12^,^13^,^14^. These data sets provide an excellent reference for paleoenvironmental reconstructions in the region. However, detailed studies of sediment color records from Miocene deposits in the northeastern Tibetan Plateau are rare, and their potential as a paleoclimate proxy require further study. Here, we examine sedimentary records from two parallel outcrop sections in the Yumen Basin (a sub-basin of the Hexi Corridor Basin) on the northeastern edge of the Tibetan Plateau (Fig. 1). Sediment redness decreased sharply at ~16.5 Ma, indicating a significant decrease of hematite production. We attribute this change to fast sediment transport, climatic cooling and wetting associated with rapid uplift of the Qilian Mountains.

## Geological and Geographical Setting

**Figure 1:**
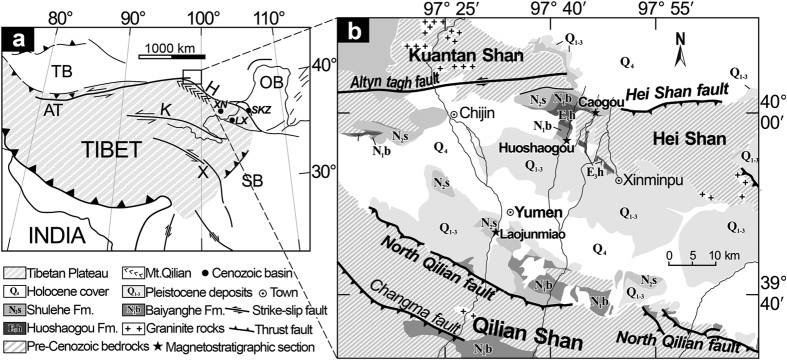
(a) Map showing the Tibetan plateau, Qilian Shan, Yumen basin and locations of sites mentioned in text. AT: Altyn tagh fault, X: Xianshuihe fault, K: East Kunlun fault, H: Haiyuan fault, XN: Xining Basin, LX: Linxia Basin and SKZ: Sikouzi Basin. **(b)** Geologic map of the Yumen Basin and locations of the Caogou (CG), Huoshaogou (HSG) and Laojunmiao (LJM) stratigraphic sections within the Yumen Basin. Figure 1 was created by CorelDRAW X7 (http://www.coreldraw.com/us/pages/free-download).

The Yumen Basin is located on the northern flank of the Qilian Shan (“Shan” means mountain, Fig. 1a,b), with elevations from 2100 m adjacent to the piedmont of the range to 1300 m in the central part of the basin. The basin is interpreted as a structurally-complex foreland depression caused by growing of the Qilian Shan. It is bounded on the south by the North Qilian Shan Fault, the Altyn Tagh strike-slip Fault to the west and the Hei Shan Fault to the north (Fig. 1b). More than 1000 m of terrigenous sediment accumulated in the basin since the late Oligocene, as indicated by paleomagnetic investigations from the Caogou (CG) and Laojunmiao (LJM) sections^15^,^16^. Fluvial and lacustrine deposits dominate the Yumen basin fill, which has been divided into five stratigraphic units (in upward sequence): the Huoshaogou, Baiyanghe and Shulehe Formations and the Yumen and Jiuquan conglomerates^17^.

**Figure 2:**
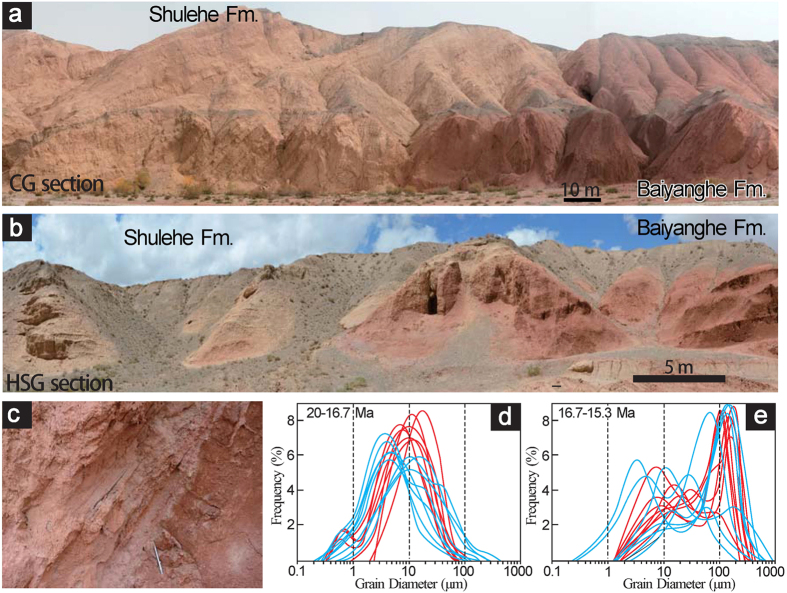
Photographs of CG and HSG sections and grain-size spectra of the fine sediments outcrop along both sections. **(a)** Photograph of the upper Baiyanghe Fm. and lower Shulehe Fm. in the CG section showing significant color change from red to khaki near the boundary of the Baiyanghe and Shulehe Formations. **(b)** View to the west of the upper Baiyanghe Fm. and lower Shulehe Fm. in the HSG section with corresponding color change in (**a**). **(c)** Cross-bedded coarse-grained sandstone in the base of the Shulehe Fm. along the CG section, exhibiting erosional contact with centimeters of relief. The pencil is 14 cm long for scale. **(d,e)** Grain-size spectra of the pre-16.5 Ma and post-16.5 Ma sediments, respectively. Blue is from the CG section and red is from the HSG section. The photographs in Fig. 2 were taken by authors.

Our study focuses on the upper part of the Baiyanghe Formation and the lower part of the Shulehe Formation along the CG and the Huoshaogou (HSG) sections, which are two parallel sections separated by ~8 km in the north part of the basin (Fig. 1b). In both sections, the Baiyanghe Formation is predominately comprised of reddish mudstone beds, which show massive structure with no bedding or distinctive laminations (Fig. 2a,b). The lower part of the Shulehe Formation is characterized by thick yellowish mudstone, siltstone and fine- to coarse-grained sandstone beds. Sandstones within the lower Shulehe Formation are decimeters to meters thick and are lenticular in shape with erosional bases (Fig. 2c). The sandstones are usually cross-stratified and show fining-upward sequences with basal granules and pebbles. These units may represent channelized and sheet flows in a fluvial setting.

Climatically, the Yumen Basin lies in the transitional belt of the East Asian monsoonal semi-humid areas and the northwest arid areas. At present, the mean annual temperature in the study area is 7.4 °C, with a July average of 21.6 °C and a January average of −8.8 °C. Mean annual precipitation is 98 mm, and over 70% of the precipitation falls during June to September, with a peak monthly mean rainfall of 23 mm in July.

## Results

**Figure 3:**
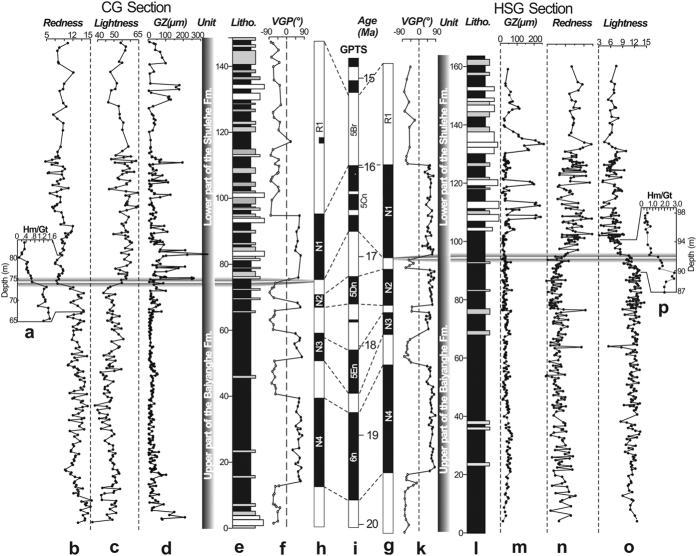
Magnetostratigraphy, color parameters, ratio of hematite to goethite and mean grain size of the CG and HSG stratigraphic sections. **(a**–**h)** Profiles of Hm to Gt ratio, redness, lightness, mean grain size (GZ), lithology, virtual geomagnetic pole latitudes (VGP) and polarity zonations of CG section. **(i)** Reference geomagnetic polarity timescale (GPTS). **(g**–**p)** Polarity zonations, VGP, lithology, GZ, lightness, redness and ratio of Hm to Gt profiles of the HSG section. Figure 3 was created by CorelDRAW X7 (http://www.coreldraw.com/us/pages/free-download).

The depositional ages of the upper part of the Baiyanghe Formation and the lower part of the Shulehe Formation from the CG section are constrained by published magnetostratigraphy results^16^. For the HSG section, we constrain the depositional ages using paleomagnetic dating. Similar to the CG section, the HSG section shows a pair of long reversed and long normal polarity zones (R1 and N1) in the lower Shulehe Formation, and two moderately long normal polarity zones (N2 and N3) and a remarkably long normal polarity zone (N4) as well as a reversed polarity zone (R5) in the upper Baiyanghe Formation (Fig. 3g,k). The CG section constrains the upper Baiyanghe Formation to the early Miocene and the lower Shulehe Formation to the middle Miocene^16^, thus we correlate the long normal polarity zones N4 and N1 to C6n and C5Cn of the geomagnetic polarity timescale (GPTS)^18^, respectively (Fig. 3i–l). The moderately-long normal polarity zones of N3 and N2 and long reversed polarity zone R1 can then be straightforwardly correlated to C5En, C5Dn and C5Br in the GPTS. Our observed magnetic polarity demonstrates that the upper part of the Baiyanghe Formation and the lower part of the Shulehe Formation were deposited between about 20 Ma and 15.3 Ma, consistent with the CG section results (Fig. 3).

We quantified sediment color and grain size by redness (a*) and lightness (L*) and grain size analysis, respectively. As shown in Fig. 3, all samples from both sections have positive a* values ranging from 3.21 to 15.22 and L* values varying from 36.95 to 64.65 (Fig. 3b,c,n,o). Along the CG and HSG sections, the a* and the L* values change sharply at ~16.5 Ma (Fig. 3f–k). The sediments before ~16.5 Ma are consistently characterized by higher a* values varying from 9.43 to 15.22, with averages of 10.2 and 12.3 in the CG and HSG sections, respectively. Lower L* values are observed for this interval, ranging from 36.95 to 61.1, with average values of 47.8 in the CG section and 46.3 in the HSG section, respectively (Fig. 3b,c,n,o). Since ~16.5 Ma, the a* value decreases distinctly to 3.21–10.83 (averaging 7.83 in the CG section and 6.45 in the HSG section), but the L* value increases dramatically to 46.0–64.65, with averages of 56.1 and 54.4 in the CG and HSG sections, respectively (Fig. 3b,c,n,o). These data clearly indicate that before ~16.5 Ma, sediments found in the studied sections are redder and darker than those after ~16.5 Ma (Fig. 2a,b). The calculated hematite (Hm) to goethite (Gt) ratio based on the second derivative of the Kubelka-Munk remission function (F(R) = (1 − R)^2^/2R)^19^,^20^) are also marked by a sharp decrease at ~16.5 Ma, consistent with decreasing redness (Fig. 3a,p). This observation suggests that the decrease in the ratio of Hm to Gt is the main reason for the color change.

The sediment grain-size distribution records of the CG section show different patterns before and after ~16.5 Ma. Before ~16.5 Ma, the grains are very fine, with small-amplitude fluctuations (Fig. 3d). Mean grain sizes for samples dated to ~20–16.5 Ma range from 2.2–70.8 μm, averaging 10.5 μm along the CG section (Figs 2d and 3d). By contrast, the grain-size record for sediments younger than ~16.5 Ma is marked by large-amplitude fluctuations (Fig. 3d). This is consistent with the lithofacies observations showing that mudstone beds inter-bedded with sandstone beds occur from 75–145 m along the CG section (Fig. 3d). For the sandstones, grain-size distributions are composed of overlapping fine and coarse components, which may represent the saltation and suspension groups. The fine component is in the grain-size range of about 1–30 μm, whereas the coarse component is within the range of 100–200 μm (Fig. 2e). This kind of grain-size distribution is very similar to modern fluvial sandstones^21^, suggesting that the sandstones were deposited in a fluvial environment. In the HSG section, the grain-size distribution patterns changed at ~16.3 Ma, slightly later than the CG section. The pre-16.3 Ma strata are uniform mudstones with mean size of 34.9 μm, and the fluvial sandstones, which consist of suspension group (1–40 μm, Fig. 2e) and saltation group (80–200 μm, Fig. 2e) appear at 16.3 Ma (Fig. 3l,m). Together with the field observations, our grain size distributions suggest that the paleoenviroment changed from shallow lacustrine to fluvial at ~16.5 Ma in the CG section and at ~16.3 Ma in the HSG section (Fig. 3d–m).

## Discussion

Color changes from reddish to yellowish are one of the most striking features of the late Cenozoic fluviolacustrine sediments in the northeastern Tibetan Plateau and the aeolian sequences on the Chinese Loess Plateau. Redness in fine-grained sediment and soils is affected primarily by fine-grained hematite (Hm) produced during weathering^13^,^22^,^23^. Generally, the redder sediments are, the higher concentration of fine Hm or the higher ratio of Hm/Gt. The key question for interpreting sediment color change, especially redness change, is the relationship between the concentrations of Hm and Gt in the fine-grained sediments. Schwertmann^24^ suggested that Hm is favored by warmer and drier climate, whereas Gt is favored by cooler and wetter climate, although the pH and organic matter in soils can also affect the distribution of Hm and Gt^25^. Recent studies improve understanding of Hm formation significantly. Based on studies of the loess-paleosol sequences on the Chinese Loess Plateau^20^,^26^ and the late Quaternary soils on Hainan Island, workers proposed low- and high-rainfall thresholds that control the formation of Hm and Gt. Above the low rainfall threshold, Hm is favored; with increasing rainfall, the concentration of Hm and ratio of Hm to Gt increase because the wetting-drying cycles characterized by monsoonal climate are suitable for amorphous iron ion to transform to ferrihydrite, a precursor to Hm, instead of Gt. With a further increase in rainfall (over the high threshold of ~600 mm/yr and temperatures around 10 °C), Hm is no longer produced. Amorphous iron ion will not transform to ferrihydrite and instead transforms into Gt. Hm formation in these Quaternary soils occurs when increasing precipitation is coupled with warmer climate. However, during the geological past, temperature and precipitation trends may be decoupled^27^,^28^, so the precipitation threshold values of these Quaternary soils might not necessarily be applied to soils during other time intervals. Furthermore, Hm content in soils is also related to duration of weathering. For example, the red clay sediments dating to 4.8–4.1 Ma have the reddest color in the red clay sequence on the Chinese Loess Plateau, and this interval corresponds to the lowest dust accumulation rates of the entire red clay sequence^27^. Nie *et al*.^27^ proposed that the reddest color during this interval does not indicate climate optimum but instead results from prolonged weathering.

With these developments and complications in mind, we attribute decrease in redness of the studied sections to three factors: fast sediment transport (and thus shortened weathering duration), climatic wetting, and regional cooling. These dramatic climate changes are direct evidence for a phase of rapid uplift of the Qilian Shan at ~16.5 Ma. We rule out grain size variation as the cause for color changes because the redness decrease in the HSG section occurrs ~200 kyr earlier than the grain size increase, and mudstone after 16.5 Ma has significantly decreased redness in comparison with the mudstone before this time (Figs 2b and 3l,m). We also rule out the possibility that climate drying caused redness decrease because the color change occurs within the middle Miocene climate optimum (~17–14 Ma) and it is not likely that climate drying would occur during this extremely warm period without tectonic activities. Indeed, because the studied sections are located to the windward side of the moisture source, uplift of the Qilian Shan would cause climate wetting and cooling instead of drying.

Actually, during the early Miocene, basin provenance and paleoenvironment reconstruction studies^29^ demonstrate relatively low mountain relief around the Hexi Corridor Basin. This low relief gave rise to sustained, slow denudation rates, resulting in fine sedimentation and low production of clastic particles. Under such slow denudation rates, the source materials undergo prolonged *in situ* chemical weathering. Moreover, it also takes more time to transport the erosive products into the basin by low-energy river systems that would have developed in the low relief of the mountain belts. Both of these processes would prolong soil formation and cause relatively high concentrations of Hm to be present within the fine grained sediments.

Middle Miocene rapid uplift of the Qilian Shan should create high relief in the south basin margins and increase bedrock erosion rates in response to high-energy release. The elevated Qilian Shan can also provide more potential energy to rivers to transport the erosive products into the basin quickly. As a result, the soil formation duration could be significantly shortened, causing reduction in Hm production. Therefore, middle Miocene sediments accumulated in the Yumen Basin with decreased concentrations of Hm and less redness, similar to the Miocene sediment records from the Xining and the Sikouzi basins of northeastern Tibet.

Moreover, a sporopollen record from the LJM section in the south part of the Yumen Basin (Fig. 1) reveals an increasing trend of humidity in the middle and late Miocene time^30^.

Outside the Qilian Shan, redness of Cenozoic sediments in the Xining Basin decreased significantly after 19.7 Ma, similar to the timing of rapid uplift of the Laiji Shan^31^, present at the southern edges of the Xining Basin in the northeastern Tibetan Plateau. Much farther northeast, decrease in redness and increase in lightness of Cenozoic deposits preserved in the Sikouzi Basin occurred at ~12 Ma^32^, coeval with the rapid uplift of the Liupan Shan at ~11 Ma^33^,^34^. The sum of these data indicates that the Miocene sediment color changes in northeastern Tibet basins are closely related to marginal mountain building during corresponding time intervals. On the other hand, Miocene sediment color changes in the northeastern Tibetan basins did not occur simultaneously, which in turn suggests that Miocene climate change, as documented by decreased redness and increased lightness of sediments, may result from the uplift of mountain ranges in the northeastern Tibetan Plateau.

## Conclusion

The Miocene deposits of the Yumen Basin provide rich records of central Asian climate change and surface uplift of the northeastern Tibetan Plateau. Our magnetostratigraphy, sediment color and grain-size data sets from the basin show that the climate shift (from the warmer and drier to colder and wetter conditions) occurred at ~16.5 Ma, as indicated by the increase in lightness (L*), decrease in redness (a*) and changing hematite to goethite rate. Comparisons of the middle Miocene climate change in the Yumen Basin with possible causal mechanisms reveals that the middle Miocene climate change in the study region likely results from the rapid uplift of the Qilian Shan in the northeastern Tibetan Plateau. After this, the uplift of the Qilian Shan caused the middle Miocene cooling and further wetting in the windward locations along the northeastern margin of the Tibetan Plateau.

## Method

To better constrain the existing record in time, a total of 152 oriented magnetostratigraphic specimens were taken from the 160 m thick HSG section to correlate with the CG section. All of the samples were subjected to stepwise thermal demagnetization from 20 °C to 640–680 °C with 10–50 °C temperature increments. The magnetic remanence was measured with a three-axis, 2G cryogenic magnetometer at the Paleomagnetism Laboratory of the Institute of Geology and Geophysics of the Chinese Academy of Sciences, where all equipment is shielded in a geomagnetic field-free space (background field <300 nT).

Principal component analysis^35^ was used to determine characteristic remanent magnetization (ChRM) only on the high-temperature component (above 450 °C). Specimens with no separated ChRM directions or with maximum angular deviation of ChRM directions >15° were rejected from further analyses. Finally, a total of 137 samples were used to obtain the magnetic polarity of the HSG section. These samples pass the C quality reversal test of McFadden and McElhinny^36^ and Tauxe and Watson’s^37^ fold test as described by Wang *et al*.^16^. The paleomagnetic correlation for the HSG section is constrained by mammal assemblages in the underlying HSG Formation^38^ and regional lithofacies correlations^16^.

A total of 194 and 310 color samples were collected from the CG and HSG sections, respectively. These samples were dried at 40 °C for two days and then were polished to expose a fresh plane larger than 3 cm^2^ for color measurement. The color reflectance was measured along the polished planes by using a handheld Miniscan EZ spectrophotometer. The color reflectance of all samples are given by the spherical L*a*b* color space. The Lightness (L*) and the redness (a*; +a* is the red direction, −a* is the green direction) chromaticity were used as proxy for sedimentary color in both sections. The analytical color reference of the CG and HSG sections are in supplemental tables 1 and 2. In addition, 25 mudstone samples were collected from the CG section within 15 meters, where sedimentary color significantly changed, and another 25 samples were collected from the HSG section in the corresponding place. The visible diffuse reflectance spectrum (DRS) serves to characterize iron oxides (especially hematite and goethite) in these 50 samples. The concentrations of hematite and goethite usually have been estimated by appropriate standards and different parameters derived from the spectrum of the reflectance (R) itself but also the spectra of the absorbance (A = log 1/R) or the Kubelka-Munk (K-M) remission function F(R) = (1 − R)^2^/2R)^19^,^20^. In this paper, the second derivative of the K-M remission function was applied to calculate the hematite to goethite rate.

Grain-size samples were taken from the CG and HSG sections at 0.2 m to 1 m intervals and the samples were analyzed using a Malvern Master Sizer 2000, with a size detection range of 0.02–2000 μm. The details of the procedure are described by Fan *et al*.^39^.

## Additional Information

**How to cite this article**: Wang, W. *et al*. Uplift-driven sediment redness decrease at ~16.5 Ma in the Yumen Basin along the northeastern Tibetan Plateau. *Sci. Rep.* **6**, 29568; doi: 10.1038/srep29568 (2016).

## Supplementary Material

Supplementary Information
