# Supplementary material for: Uplift-driven sediment redness decrease at ~16.5 Ma in the Yumen Basin along the northeastern Tibetan Plateau
Source: Sci Rep. 2016 Jul 14;6:29568. doi: 10.1038/srep29568 (PMC4944177; doi:10.1038/srep29568)
Supplement: Supplementary Information [file srep29568-s1.pdf]

Uplift-driven sediment redness decrease at ~16.5 Ma in the Yumen Basin along the northeastern Tibetan Plateau

Weitao Wang[1], Peizhen Zhang[1,2], Wenjun Zheng[1], Dewen Zheng[1], Caicai Liu[1], Hongyan Xu[1], Huiping Zhang[1], Jingxing Yu [1], Jianzhang Pang[1]

[1]State Key Laboratory of Earthquake Dynamics, Institute of Geology, China Earthquake Administration, Beijing, China

[2]School of Earth Science and Geological Engineering, Sun Yat-sen University, Guangzhou, China

Fig.s1 Orthogonal (Zijderveld) vector plots of the representative thermal demagnetization behaviors of specimens from the Huoshaogou (HSG) section.

Fig.s2 Equal-area plots of accepted characteristic remanent magnetization directions ChRMs (137 sites) from the HSG section.

Fig.s3 Positive fold test for the HSG's ChRMs.

Table s1. Color reference for the CG section.

Table s2. Color reference for the HSG section.

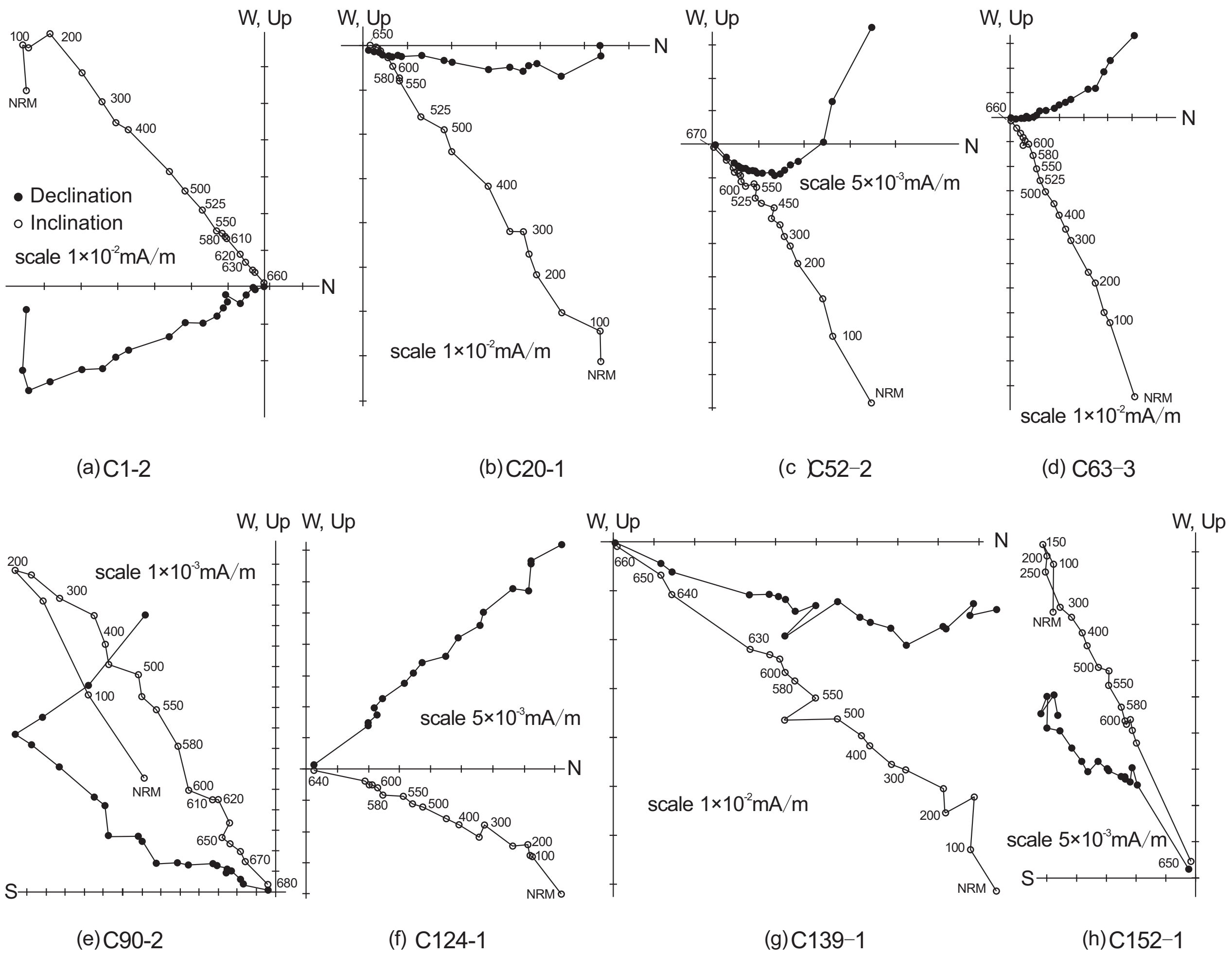

Fig.s1 Orthogonal (Zijderveld) vector plots of the representative thermal demagnetization behaviors of specimens from the Huoshaogou (HSG) section. The hollow (solid) circles show the declination (inclination) within thethe orthogonal demagnetization diagrams. NRM is the natural remanent magnetization before demagnetization, and the numbers mark the temperature steps of demagnetization. The representatie demagnetiization diagrams (a-h) illustrate high-quality demagnetization behaviour after removing a secondary viscous remanent magnetization at 150–200°C

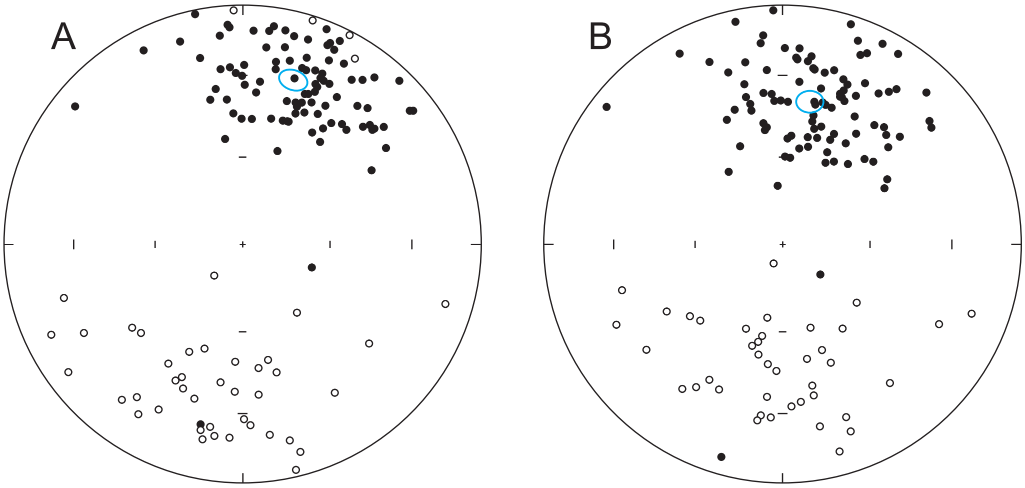

Normal
Dg=19.0, Ig=26.9, α95= 12.6, N= 96
Reversed
Dg=192.2, Ig= -33.6, α95= 7.03, N= 41.

Normal
Ds=13.3, Is=37.5, α95= 11.9, N= 96
Reversed
Ds=184.2, Is=-43.5, α95= 7.20, N= 41.

Fig. s2 Equal-area plots of accepted ChRMs (137 sites) from the Huoshaogou (HSG) section in (A) geographic and (B) tilt-corrected coordiniates. The hollow (solid) circles plot in the lower (upper) hemisphere. The ovals indicate the α95 error around the Fisher's mean with the mean data. The difference of 9.1° between the overall mean normal and reversed polarity directions and a calculated critical angle of 9.8° suggest that the HSG magnetostratigraphy passes the B quality reversal test.

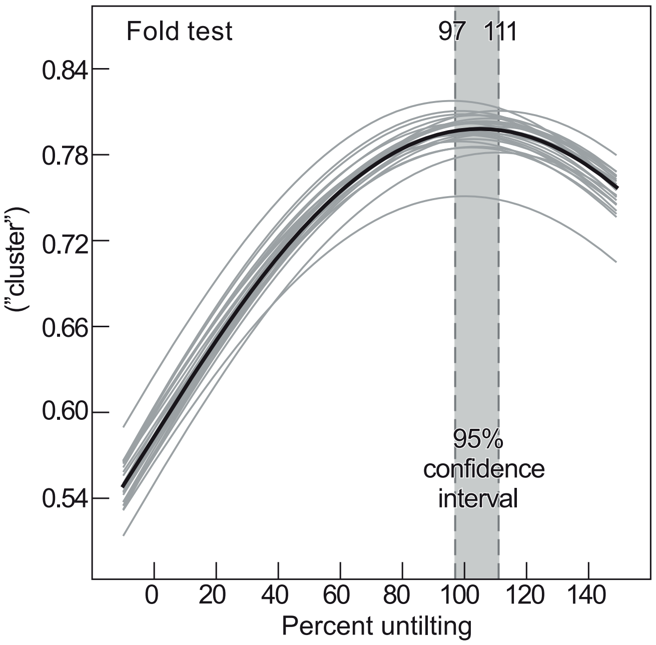

Fig. s3 Positive fold test for HSG's characteristic remanent magnetization (ChRM) directions shown by the 95% confidence interval of parameter of cluster, which overlaps with the 100% untilted ChRM vectors after correcting for the stratum orientation. The cluster maximum reflects the tightest grouping of the ChRM directions during the progressive untilting of the strata and is defined by the eigenvalues of the orientation matrix.

Table s1 Color reference of the CG section, the unit of depth is in meter.

Sample ID	L*	a*	b*	depth	Sample ID	L*	a*	b*	depth
CB1	36.95	12.14	15.22	0.40	CB43	48.43	10.29	15.21	28.70
CB2	49.08	13.65	20.28	1.20	CB44	42.22	13.49	17.19	29.20
CB3	47.40	13.73	19.64	1.70	CB45	47.58	12.46	17.73	30.10
CB4	45.69	13.24	19.30	2.80	CB46	42.87	13.04	16.75	31.20
CB5	51.23	14.35	21.79	3.30	CB47	47.16	10.16	15.46	32.00
CB6	47.57	12.73	19.07	4.30	CB48	41.05	12.96	14.51	32.30
CB7	51.06	14.92	21.89	5.30	CB49	45.23	13.48	15.87	32.70
CB8	48.50	13.90	20.31	5.80	CB50	46.17	11.03	14.26	33.50
CB9	50.47	13.75	20.05	6.10	CB51	41.19	12.28	16.04	34.00
CB10	50.65	14.16	21.19	6.50	CB52	40.16	13.22	15.96	35.00
CB11	51.27	12.91	19.72	7.00	CB53	47.30	11.65	16.09	35.50
CB12	42.67	15.22	19.06	7.30	CB54	45.63	13.15	17.38	36.00
CB13	42.28	12.14	15.47	8.80	CB55	38.54	14.26	16.53	36.40
CB14	44.31	12.22	16.92	9.30	CB56	47.28	12.81	17.73	37.50
CB15	49.69	11.12	16.23	9.50	CB57	47.98	13.23	18.51	37.90
CB16	50.04	12.25	18.26	10.00	CB58	48.53	10.92	16.18	38.70
CB17	53.59	11.99	16.97	10.70	CB59	46.15	11.29	14.50	39.00
CB18	42.96	13.20	17.10	11.00	CB60	43.89	14.13	16.02	40.00
CB19	48.83	11.58	14.29	11.50	CB61	47.32	10.24	14.68	40.40
CB20	49.23	11.78	16.01	12.00	CB62	46.28	11.30	15.47	41.00
CB21	51.46	11.26	17.61	12.70	CB63	44.34	13.19	16.93	41.40
CB22	50.91	11.40	18.52	13.10	CB64	44.53	12.77	15.11	42.00
CB23	50.52	12.49	18.19	14.20	CB65	46.24	13.47	18.46	42.40
CB24	49.29	11.45	17.05	14.50	CB66	49.60	13.66	19.92	43.70
CB25	53.10	10.41	13.17	15.40	CB67	44.06	12.52	17.08	44.50
CB26	52.62	10.61	14.78	15.80	CB68	44.48	13.51	16.05	45.00
CB27	51.98	9.35	15.38	16.40	CB69	45.37	12.18	17.61	45.40
CB28	49.20	9.21	14.28	16.80	CB70	43.45	11.83	14.12	45.70
CB29	50.09	10.94	15.34	18.20	CB71	43.33	11.51	13.66	46.00
CB30	49.16	10.70	14.96	18.70	CB72	41.90	12.04	16.29	46.60
CB31	47.65	11.31	15.06	19.50	CB73	50.19	13.27	19.12	47.00
CB32	48.20	11.15	15.27	19.80	CB74	54.26	12.24	18.00	47.40
CB33	49.34	11.55	15.76	20.70	CB75	45.50	12.00	14.49	47.80
CB34	49.15	11.52	15.53	21.10	CB76	49.30	11.16	17.85	48.20
CB35	50.77	12.37	18.48	21.60	CB77	47.34	11.41	15.54	48.50
CB36	46.92	10.52	14.05	22.30	CB78	46.71	9.81	14.66	48.90
CB37	49.36	10.59	15.25	22.80	CB79	47.24	9.98	14.96	49.10
CB38	49.34	11.18	15.56	24.00	CB80	50.83	10.70	16.88	50.10
CB39	46.92	10.99	14.96	24.60	CB81	45.54	11.97	14.79	50.50
CB40	44.55	12.63	15.43	25.50	CB82	49.90	11.30	16.42	51.30
CB41	48.69	11.31	16.24	27.00	CB83	43.64	14.16	15.88	51.70
CB42	50.10	12.10	17.28	27.50	CB84	47.16	10.78	14.46	52.40

Samp. ID	L*	a*	b*	Depth	Samp. ID	L*	a*	b*	Depth
CB85	48.42	13.07	15.64	53.00	CS15	54.37	8.14	19.12	83.30
CB86	52.61	11.63	17.81	54.10	CS16	60.87	8.92	20.72	84.40
CB87	53.32	12.49	18.45	54.50	CS17	56.77	8.70	20.95	84.80
CB88	48.38	11.93	17.31	56.00	CS18	60.69	9.11	21.22	86.50
CB89	55.49	10.78	21.22	56.80	CS19	59.57	8.57	22.62	86.60
CB90	52.82	11.18	17.14	58.30	CS20	59.50	8.65	21.20	86.90
CB91	50.07	11.07	17.02	59.60	CS21	56.91	8.42	17.28	87.70
CB92	48.68	12.82	16.06	59.80	CS22	58.45	9.59	21.81	88.40
CB93	54.40	11.82	19.10	60.00	CS23	56.70	9.38	21.41	88.70
CB94	50.46	11.02	16.95	60.80	CS24	49.63	9.95	18.17	89.10
CB95	52.91	12.07	19.49	61.50	CS25	54.54	9.23	17.96	89.50
CB96	48.93	12.46	15.82	61.90	CS26	58.21	10.46	23.07	89.80
CB97	46.00	13.49	18.23	62.50	CS27	57.04	10.71	22.75	90.20
CB98	48.17	12.63	16.07	62.80	CS28	55.70	10.97	21.65	91.80
CB99	48.73	11.06	18.38	63.40	CS29	54.57	8.21	18.63	92.30
CB100	53.29	9.78	17.51	63.80	CS30	57.14	8.97	20.29	92.70
CB101	48.98	11.18	17.64	64.50	CS31	58.71	8.69	22.79	94.50
CB102	45.99	12.85	15.67	64.90	CS32	53.62	8.43	15.75	96.00
CB103	49.26	13.42	19.74	65.40	CS33	57.51	6.91	16.86	96.40
CB104	47.33	13.36	17.41	65.70	CS34	63.28	6.57	18.95	97.30
CB105	49.91	12.08	21.17	67.20	CS35	64.18	6.32	19.75	97.60
CB106	48.53	10.49	17.58	68.30	CS36	56.81	6.95	17.07	98.00
CB107	49.10	11.14	19.72	68.70	CS37	57.94	7.37	18.45	98.30
CB108	45.22	12.89	19.21	69.60	CS38	57.28	7.39	16.82	98.60
CB109	46.94	12.26	19.04	70.10	CS39	57.97	8.15	19.10	99.10
CB110	50.68	12.07	18.21	70.60	CS40	59.27	8.14	19.28	100.70
CB111	43.63	13.39	17.88	71.00	CS41	56.98	7.40	17.50	101.00
CB112	46.43	10.32	20.58	72.50	CS42	64.25	5.77	16.08	101.90
CB113	47.74	11.13	21.44	72.90	CS43	57.05	7.32	19.34	102.30
CS1	54.65	7.51	20.67	74.10	CS44	46.96	8.37	15.88	103.00
CS2	54.32	7.31	17.93	75.00	CS45	51.22	9.97	16.83	103.40
CS3	57.32	7.88	20.39	75.40	CS46	57.64	6.36	16.82	104.40
CS4	56.88	7.58	16.38	76.20	CS47	57.45	7.75	18.69	104.70
CS5	57.81	7.95	17.04	76.50	CS48	59.56	7.42	20.25	105.50
CS6	52.70	7.84	18.30	76.90	CS49	53.00	8.16	18.13	106.10
CS7	57.91	8.58	17.90	77.20	CS50	52.29	8.91	22.84	106.40
CS8	53.53	8.74	20.70	78.10	CS51	54.84	8.53	17.85	107.70
CS9	50.81	8.67	19.64	78.50	CS52	57.56	5.31	14.49	108.10
CS10	57.71	8.24	18.59	79.40	CS53	62.06	5.58	13.87	108.30
CS11	59.90	6.98	18.00	80.20	CS54	55.08	8.54	18.00	108.60
CS12	59.90	6.99	18.00	80.60	CS55	59.32	9.17	21.99	109.30
CS13	56.18	7.38	18.66	81.30	CS56	58.20	8.76	19.97	110.10
CS14	58.93	8.55	22.22	82.80	CS57	56.44	6.15	15.12	110.70

Sample ID	L*	a*	b*	depth
CS58	50.53	7.52	15.98	111.10
CS59	60.72	7.16	16.14	111.20
CS60	63.13	4.73	15.20	111.70
CS61	48.29	6.27	17.22	112.20
CS62	55.73	7.94	22.70	112.70
CS63	59.94	7.04	20.12	113.00
CS64	51.06	7.60	14.86	113.30
CS65	55.11	7.47	19.26	114.70
CS66	58.52	8.16	18.92	116.10
CS67	55.09	8.69	19.47	121.20
CS68	55.50	8.79	22.81	123.40
CS69	53.39	7.45	19.37	124.80
CS70	53.26	6.55	14.24	126.10
CS71	52.80	9.45	19.42	128.50
CS72	46.41	8.76	16.98	129.60
CS73	48.29	7.98	18.85	133.40
CS74	50.74	8.73	20.58	133.80
CS75	51.75	8.96	16.13	135.60
CS76	47.74	11.05	19.50	139.00
CS77	54.85	7.70	18.47	141.90
CS78	53.05	7.63	17.67	142.20
CS79	56.66	7.50	19.07	145.70
CS80	56.96	7.92	18.60	146.70
CS81	54.00	9.83	22.50	148.10

Table s2. Color reference of the HSG section. The unit of depth is in meter

Sample ID	L*	a*	b*	depth	Sample ID	L*	a*	b*	depth
HB1	46.5	12.6	16.36	2.3	HB43	48.7	10.38	15.05	33.6
HB2	53.43	11.77	18.35	4.1	HB44	44.04	11.21	14.49	34.4
HB3	42.03	10.96	14.13	5.5	HB45	43.95	11.17	14.46	34.5
HB4	53.16	10.95	16.76	7.1	HB46	50.02	12.11	18.23	34.6
HB5	47.34	11.68	15.72	8.7	HB47	49.91	10.04	13.5	35.4
HB6	48.39	11.25	16.68	9.0	HB48	49.16	8.51	11.75	35.8
HB7	52.61	9.04	13.6	10.2	HB49	43.61	12.65	14.26	38.2
HB8	55.7	9.05	15.32	10.5	HB50	45.15	11.85	14.36	39.6
HB9	44.71	11.43	13.87	11.5	HB51	49.11	12.35	17.18	39.9
HB10	52.13	10.67	16.71	11.9	HB52	46.86	12.45	15.95	40.5
HB11	51.22	10.33	16.7	13.8	HB53	43.95	12.52	14.41	40.7
HB12	42.91	10.53	13.43	14.1	HB54	47.79	10.8	14.07	41.3
HB13	48.31	11.16	16.64	14.9	HB55	50.26	11.83	16.33	41.4
HB14	49.89	11.09	15.17	15.2	HB56	43.81	9.32	11.4	41.5
HB15	48.37	10.45	15.28	17.2	HB57	43.17	12.83	14.82	41.8
HB16	44.47	11.47	13.79	17.6	HB58	47.83	13.31	17.49	42.0
HB17	43.79	11.28	13.6	19.4	HB59	45.5	11.13	13.55	42.6
HB18	54.02	12.24	19.38	19.7	HB60	45.45	11.53	14.68	43.0
HB19	45.66	11.2	13.85	20.3	HB61	46.05	11.14	13.73	43.4
HB20	53.1	11.36	17.48	20.7	HB62	49.61	12.16	16.87	43.7
HB21	46.9	11.06	13.47	21.3	HB63	47.93	12.29	15.55	44.1
HB22	46.29	11.14	15.67	21.7	HB64	50.15	11.73	15.18	44.4
HB23	46.88	11.14	14.01	22.0	HB65	43.69	10.5	13.11	45.2
HB24	48.29	12.22	17.93	22.4	HB66	46.26	10.98	15.29	45.6
HB25	44.68	11.74	14.14	22.9	HB67	45.26	12.46	14.65	46.3
HB26	52.37	11.88	17.59	23.5	HB68	48.76	11.8	15.87	46.6
HB27	42.73	11.6	13.43	23.9	HB69	44.25	11.97	14.38	47.2
HB28	54.15	12.23	18.99	24.2	HB70	47.27	11.08	15.17	47.6
HB29	49.76	10.39	14.46	25.0	HB71	46.12	11.4	13.53	48.1
HB30	52.2	9.61	15.78	25.4	HB72	45.04	10.08	12.85	48.5
HB31	44.57	11.54	13.54	26.3	HB73	43.88	13	14.35	49.1
HB32	52.4	10.52	16.59	26.7	HB74	44.43	11.68	14.46	49.6
HB33	45.35	12.24	14.78	27.5	HB75	44.28	11.23	13.36	50.3
HB34	50.37	9.85	14.93	27.8	HB76	44.5	10.57	14.15	50.6
HB35	48.6	10.88	13.97	29.4	HB77	46.44	12.65	16.15	51.0
HB36	48.29	10.05	15.21	29.8	HB78	42.87	11.41	13.64	51.4
HB37	49.37	12.62	16.9	30.4	HB79	39.5	11.25	11.8	51.7
HB38	46.78	10.53	14.72	30.7	HB80	40.14	11	13.72	52.0
HB39	42.1	13.12	15.09	31.4	HB81	49.94	10.89	15.19	52.2
HB40	43.77	13.44	15.52	32.4	HB82	46.28	11.26	14.48	52.8
HB41	50.55	12.19	17.57	32.7	HB83	41.71	13.22	15.14	53.5
HB42	47.99	11.49	14.86	33.2	HB84	46	12.42	15.73	53.9

Sample ID	L*	a*	b*	depth	Sample ID	L*	a*	b*	depth
HB85	44.82	11.68	14.66	54.6	HB128	49.01	12.35	16.58	74.6
HB86	48	11.03	14.91	55.0	HB129	46.02	11.69	14.18	74.8
HB87	43.33	12.8	15.29	55.3	HB130	42.02	11.17	12.26	74.9
HB88	47.6	10.89	15.59	55.7	HB131	43.95	11.29	13.79	75.0
HB89	41.57	12.43	14.02	56.7	HB132	42.8	13.48	15.06	75.3
HB90	46.14	12.07	15.69	57.2	HB133	45.56	12.5	15.87	75.7
HB91	47.11	12.13	14.89	58.4	HB134	42.4	10.92	11.92	75.8
HB92	44.27	9.98	12.48	58.9	HB135	45.01	11.43	14.3	75.9
HB93	45.16	10.99	14.37	59.4	HB136	42.1	10.28	11.93	76.0
HB94	45.52	12.51	15.29	59.6	HB137	44.25	12.73	14.52	76.1
HB95	46.96	11.54	15.16	59.9	HB138	41.52	10.82	12.78	76.2
HB96	46.73	12.16	15.19	60.4	HB139	41.6	13.6	16.79	76.5
HB97	44.34	8.68	11.63	61.4	HB140	44.6	14.42	16.95	76.7
HB98	58.81	5.4	10.27	61.7	HB141	41.51	12.46	15.31	76.9
HB99	41.92	12.58	15.4	62.2	HB142	46.29	10.03	14.03	77.0
HB100	46.73	11.71	16.32	62.6	HB143	47.89	10.81	15.2	77.3
HB101	42.02	12.49	14.05	63.3	HB144	42.36	13.02	14.36	77.5
HB102	46.18	12.24	15.63	63.5	HB145	42.04	11.69	13.75	77.9
HB103	45.43	12.31	14.6	63.9	HB146	43.23	12.74	14.97	78.1
HB104	46.72	12.21	14.95	64.0	HB147	45.86	10.5	13.59	78.4
HB105	47.98	12.67	16.46	64.1	HB148	44.94	12.83	14.77	78.7
HB106	43.59	12.1	14.65	64.5	HB149	47.06	12.5	15.89	79.2
HB107	48.3	11.49	15.78	64.9	HB150	47.74	12.55	16.03	79.7
HB108	45.94	12.82	14.54	65.7	HB151	42.92	13.63	15.56	80.1
HB109	50.09	12.36	15.54	66.0	HB152	40.4	11.02	12.33	80.6
HB110	46.12	12.11	14.4	66.6	HB153	44.25	10.9	13.9	81.0
HB111	46.61	11.35	15.45	66.9	HB154	42.67	12.16	13.87	81.5
HB112	47.84	11.57	15.39	67.7	HB155	45.46	13.48	17.34	81.7
HB113	47.56	10.29	13.72	68.1	HB156	43.01	11.99	14.73	82.1
HB114	51.27	10.51	14.22	68.7	HB157	47.44	12.98	16.89	82.4
HB115	48.68	11.88	16.42	69.2	HB158	43.5	12.71	15.53	83.1
HB116	43.44	13.18	15.11	69.7	HB159	47.88	12.45	15.94	83.4
HB117	48.02	12.94	17.11	70.0	HB160	42.78	10.7	13.38	83.8
HB118	43.42	11.96	14.1	70.4	HB161	43.8	13.27	16.44	84.2
HB119	43.97	11.8	14.36	70.6	HB162	39.85	9.87	11.61	84.5
HB120	51.57	10.89	15.83	71.0	HB163	50.07	11.53	15.29	84.7
HB121	46.33	12.37	15.7	71.5	HB164	42.53	13.13	15.77	85.1
HB122	44.18	13.16	16.88	71.9	HB165	43.23	10.8	13.29	85.4
HB123	45.8	12.16	15.56	72.3	HB166	45.88	12.74	16.07	85.6
HB124	50.71	9.51	13.93	72.5	HB167	43.58	12.32	15.15	85.8
HB125	45.04	12.06	13.86	73.2	HB168	47.32	13.33	16.55	86.1
HB126	47.82	12.37	15.74	73.6	HB169	48.13	11.71	15.77	86.3
HB127	61.16	9	12.77	74.4	HB170	46.62	11.69	14.21	86.7

Sample ID	L*	a*	b*	depth	Sample ID	L*	a*	b*	depth
HB171	44.97	12.54	15.63	86.9	HS17	48.32	7.77	14.35	99.5
HB172	43.83	12.81	15.41	87.4	HS18	56.65	8.25	20.73	99.6
HB173	45.01	13.19	16.01	87.7	HS19	56.65	8.2	20.63	99.7
HB174	45.2	12.69	14.64	88.3	HS20	55.23	3.12	14.51	99.8
HB175	44.9	11.95	15.14	88.5	HS21	50.96	9.23	21.19	100.0
HB176	43.11	10.7	13.18	88.7	HS22	56.75	5.59	14.89	100.1
HB177	46.17	12.31	15.61	88.9	HS23	48.33	9	20.83	100.2
HB178	45.53	12.15	17.53	89.2	HS24	64.65	3.84	11.72	100.5
HB179	47.32	11.07	15.57	89.4	HS25	55.49	7.38	19.79	101.3
HB180	45.09	10.87	14.24	89.7	HS26	54.14	5.51	14.06	101.6
HB181	45.37	10.34	13.75	89.9	HS27	50.98	8.15	19.03	102.5
HB182	45.93	9.35	13.02	90.1	HS28	53.83	7.14	18.49	102.7
HB183	46.83	9.26	11.84	90.2	HS29	59.14	4.26	18.96	103.1
HB184	47.77	11.25	14.64	90.8	HS30	58.87	3.57	16.66	103.3
HB185	49.46	10.53	15.46	91.0	HS31	54.62	7.2	18.61	103.8
HB186	52	10.38	15.61	91.2	HS32	55.77	6.61	17.6	104.0
HB187	46.35	9.96	13.62	91.3	HS33	51.83	8.49	17.02	104.4
HB188	40.32	10.08	12.3	91.7	HS34	48.78	6.57	13.28	104.6
HB189	47.36	11.25	17.18	92.0	HS35	49.33	7.65	13.5	104.8
HB190	49.14	10.82	18.16	92.3	HS36	54.31	7.39	20.48	105.1
HB191	44.58	9.67	13.77	92.7	HS37	58.65	6.74	19.49	105.5
HB192	49.58	10.53	16.09	92.7	HS38	64.53	3.95	11.03	105.8
HB193	50.16	9.44	16.27	93.3	HS39	61.22	3.62	11.1	106.0
HB194	49.19	9.79	17.11	93.7	HS40	59.52	6.08	15.22	106.4
HB195	51.45	10.44	18.69	94.0	HS41	48.48	8.08	17.28	106.6
HB196	56.86	10.36	20.18	95.0	HS42	57.97	6.12	15.34	106.9
HB197	46.82	9.11	14.42	95.5	HS43	55.97	7.35	17.31	107.5
HS1	56.71	8.51	19.24	97.0	HS44	58.69	6.5	16.37	107.6
HS2	50.11	8.03	15.44	97.2	HS45	59.99	6.68	16.96	108.6
HS3	50.96	7.02	14.79	97.3	HS46	52.9	7.16	18.97	109.1
HS4	52.13	8.05	17.22	97.6	HS47	50.36	3.66	10.06	109.4
HS5	55.25	5.56	12.51	97.8	HS48	56.75	4.13	11.57	109.6
HS6	52.52	8.02	17.02	98.2	HS49	48.34	5.62	15.55	110.8
HS7	53.53	5.95	12.69	98.4	HS50	57.12	4.28	12.5	111.1
HS8	51.87	6.65	13.42	98.5	HS51	45.96	7.19	14.28	112.5
HS9	51.61	5.88	14.96	98.6	HS52	47.78	7.6	14.45	113.1
HS10	56.87	5.91	15.1	98.6	HS53	53.99	6.6	14.74	114.2
HS11	55.22	6.6	14.56	98.9	HS54	52.23	6.86	13.7	114.8
HS12	55.94	5.7	14.89	99.0	HS55	52.96	8.82	16.66	115.1
HS13	56.8	6.65	16.68	99.2	HS56	52.86	7.11	13.33	115.4
HS14	60.42	4.78	12.87	99.2	HS57	50.56	8.48	15.7	115.6
HS15	55.04	6.35	15.6	99.3	HS58	49.55	7.93	13.82	116.0
HS16	57.88	7.21	17.16	99.4	HS59	46.05	6.86	13.7	116.7

Sample ID	L*	a*	b*	depth	Sample ID	L*	a*	b*	depth
HS60	46.78	7.03	12.18	117.3	HS87	53.4	7.43	18.66	126.1
HS61	63.29	3.99	11.64	118.2	HS88	48.93	8.2	17.52	126.4
HS62	52.77	4.08	11.53	118.7	HS89	56.13	7.26	15.36	127.0
HS63	53.1	8.66	16.03	119.8	HS90	53.55	7.23	17.22	127.3
HS64	54.74	5.63	12.7	120.1	HS91	56.08	7.24	15.37	127.7
HS65	61.4	4.94	15.33	120.5	HS92	53.1	7.64	15.95	128.5
HS66	46.88	7.39	15.43	120.9	HS93	49.94	9.87	17.42	128.8
HS67	47.03	6.96	15.28	121.4	HS94	58.18	7.2	15	129.8
HS68	61.18	5.87	15.79	121.8	HS95	59.64	6.41	13.87	130.2
HS69	60.39	5.13	15.68	122.1	HS96	64.4	5.55	13.4	131.4
HS70	49.25	9.41	17.46	122.3	HS97	60.46	4.4	11.01	131.9
HS71	58.07	5.91	18.43	122.5	HS98	52.21	5.81	16.05	134.4
HS72	48.69	5.15	14.38	122.8	HS99	53.23	6.26	17.05	135.2
HS73	51.38	5.97	16.83	123.0	HS100	54.25	6.4	18.37	137.9
HS74	58.52	5.56	18.23	123.4	HS101	54.7	5.77	16.67	138.4
HS75	53.77	6.19	17.71	123.7	HS102	54.66	7.91	17.61	140.1
HS76	61.67	4.38	13.87	124.0	HS103	54.12	6.24	14.2	140.6
HS77	54.29	6.64	14.47	124.3	HS104	52.72	5.89	15.61	142.8
HS78	56.16	7.02	17.95	124.7	HS105	57.87	7.28	17.03	143.7
HS79	58.07	6.23	14.36	124.9	HS106	54.59	6.99	19.96	144.0
HS80	57.42	6.97	18	125.0	HS107	55.66	6.23	17.22	146.1
HS81	59.73	5.72	15.2	125.1	HS108	53.55	5.38	14.28	146.5
HS82	53.83	6.06	16.44	125.2	HS109	52.23	6.88	19	148.8
HS83	56.18	6.3	17.55	125.3	HS110	57.22	6.79	19.91	149.9
HS84	53.59	6.86	18.16	125.5	HS111	59.34	7	19.61	151.9
HS85	52.51	7.62	18.8	125.7	HS112	56.8	6.13	16.71	152.7
HS86	52.57	7.19	18.84	125.9	HS113	53.5	7.38	19.82	158.0
